# Effective Stabilization of Cadmium and Copper in Iron-Rich Laterite-Based Geopolymers and Influence on Physical Properties

**DOI:** 10.3390/ma16247605

**Published:** 2023-12-12

**Authors:** Rachel Yanou Nkwaju, Joëlle Nadia Fekoua Nouping, Soumayah Bachirou, Tatiane Marina Abo, Juvenal Giogetti Nemaleu Deutou, Jean Noël Yankwa Djobo

**Affiliations:** Local Materials Promotion Authority, MINRESI/MIPROMALO, Nkolbikok, Yaoundé P.O. Box 2396, Cameroon; noupiming@yahoo.fr (J.N.F.N.); bachirou.soumayah@mipromalo.cm (S.B.); tatianemarinaa@yahoo.com (T.M.A.)

**Keywords:** iron-rich laterite, solidification/stabilization, heavy metals, geopolymer, structural performance

## Abstract

This study aimed to investigate the efficiency of a geopolymer binder of the type of Na-poly(ferro–silico–aluminate) as a matrix for the stabilization of heavy metals along with their effect on the development of structural performances. The artificial contamination of soil with ions was carried out and used to prepare an alkali-activated iron-rich lateritic soil binder. Further, various microstructural analyses were carried out to explain the stabilization mechanism. The stabilization efficiency was assessed by leaching tests in de-ionized water and hydrochloric acid (0.1 M, HCl). Then, the physical properties were determined to evaluate the impact of heavy metals on the structural performance of the binder. Results demonstrated that the prepared geopolymer binder has the lowest stabilization capacity in an acidic medium (low pH) than in water with high pH. However, the stabilization of Cu ions was effective at 99%, while the Cd ion is barely retained in the matrix. Firstly, the mechanism consists of chemical bonds through ion exchange with sodium of the Na-poly(ferro–silico–aluminate) network. Secondly, through physical interaction with the pore network of the matrix, the heavy metals induced structural deterioration in the geopolymer matrix with a decrease in the compressive strength and bulk density and an increase of both apparent porosity and water absorption.

## 1. Introduction

Heavy metal-contaminated wastes or soils cause severe environmental pollution issues that need to be addressed urgently to reduce their impact on wildlife [1,2]. The valorization of such soils for the manufacturing of construction and building materials has been developed as an effective and efficient route for the sustainable mitigation of their environmental impacts [3,4]. Alkaline or acid activation of aluminosilicate, also known as geopolymerization, is a process that has been used in recent years for the valorization of contaminated solid wastes or soils in construction and building [5,6,7]. Geopolymer is a semi-crystalline compound consisting of a three-dimensional network of silico–oxide (-Si-O-Si-O-), silico–aluminate (-Si-O-Al-O-), ferro–silico–aluminate (-Fe-O-Si-O-Al-O) or alumino–phosphate (-Al-O-P-O-) [8,9]. It is one of the building materials that has become more popular in recent years because of its lower CO_2_ emission during the manufacturing process [10]. The nature of the binder chemistry depends on the type of solid raw materials used, which greatly affect the final properties. This generally consists of mineral waste materials (fly ash, slag, mine tailings, bottom ash, phosphogypsum, etc.) or natural raw materials (clay, lateritic soils, volcanic ash, etc.) [11,12,13,14,15]. The stabilization mechanism in geopolymer materials can differ significantly depending on the type of heavy metals and the nature of the aluminosilicate used.

Cadmium and copper are two heavy metals that may adversely affect the reproductive health of humans, especially male fecundity, after exposure [16]. It was shown that Cu^2+^ immobilization in metakaolin-based geopolymer consists of chemical incorporation into the geopolymer matrix and was pH dependent as a large amount of Cu^2+^ was precipitated in the form of gerhardtite (low pH) or tenorite and spertiniite (high pH) [17]. Cadmium (Cd) immobilization is effective at high pH and mainly occurs through precipitation as Cd(OH)_2_ [18]. However, it can also balance the negative charge of Al tetrahedrons in the geopolymer framework. The literature reports that the host matrices investigated to immobilize solid waste containing heavy metals are mainly low or iron-free aluminosilicate consisting of fly ash, blast furnace slag, metakaolin, or a combination of at least two of them [3,17,19,20,21]. In a geopolymer binder that consists of (Ca, Na, K)-poly(silico–aluminate) or polysialate (Ca, Na, K)-(Si-O-Al-O), the Si/Al molar ratio controls the extent of geopolymerization and the solidification/stabilization efficiency of heavy metals [22]. The iron-rich aluminosilicate gives rise to a binder chemistry of type (Ca, Na, K) poly(ferro–silico–aluminate), where iron plays a similar role to aluminum [23,24,25]. Thus, such a matrix is worth investigating for the solidification/stabilization of heavy metals. The interaction of heavy metals with a geopolymer matrix depends on many factors, including the type and composition of the precursor used. Therefore, this work aims to assess the stabilization efficiency of copper (Cu) and cadmium (Cd) on a novel geopolymer matrix based on iron-rich lateritic soil. Specifically, it consists of the assessment of the leaching behavior in various environments (water and hydrochloric acid), the use of the microstructural analysis to explain the reaction mechanism of the stabilization, and their effects on the resulting binder chemistry. Further, it stresses the effect of those metals on the development of structural performance and microstructure of geopolymer.

## 2. Experimental Methods

### 2.1. Materials

The laterite soil used as aluminosilicate was collected at Emana in the center region of Cameroon. It has been characterized by the authors in their previous works and showed that its mineralogy is composed of kaolinite (34%), illite (2%), quartz (25%), anatase (1%), goethite (29%), hematite (8%), and gibbsite (2%) [25]. The major oxides in laterite are SiO_2_ (33.18 wt.%), Al_2_O_3_ (17.89 wt.%), and Fe_2_O_3_ (35.33 wt.%). The minor oxides are TiO_2_ (1.08 wt.%), MnO (0.56 wt.%), MgO (0.23 wt.%), CaO (0.09 wt.%), K_2_O (0.05 wt.%), and P_2_O_5_ (0.34 wt.%), with a loss on ignition of 11.84 wt.%. Laterite was calcined in a programmable electric furnace for 4 h with a heating/cooling rate of 5 °C/min at 700 °C. The alkaline solution was prepared by mixing an amount of sodium hydroxide solution (10 M) with sodium silicate solution having a silica modulus of three. The final activating solution consists of a mass ratio of Na_2_SiO_3_/NaOH of 2.4. The metal salts are CdCl_2_·H_2_O (201.32 g·mol; 99%) and CuSO_4_·5H_2_O (249.68 g·mol; 99%).

### 2.2. Preparation of Geopolymer Samples with Metals

The details of the different mixes are given in Table 1. Each geopolymer was prepared by first mixing calcined laterites with the alkaline solution and stirring for 3 min. Then, the paste was mixed with solid heavy metal salt for 5 min at 200 rpm. The resulting viscous paste obtained was cast into a 40 × 40 × 40 mm mold. The sample was left at room temperature for 24 h before demolding. The obtained geopolymers were sealed in plastic bags for 28 days before performing characterization.

### 2.3. Stabilization Efficiency on Iron-Rich Lateritic Soil-Based Geopolymer

The stabilization efficiency was assessed by performing the leaching behavior of the contaminated geopolymers in distilled water (pH = 6.96) and hydrochloric acid (HCl) with a concentration of 0.1 M (pH = 1.08) as a leaching medium. The previously prepared 40 × 40 × 40 mm samples were cut into small pieces of 40 × 40 × 2 mm to carry out the leaching tests. The samples were immersed in the leaching solution and stirred for 24 h on a shaking table. Then, the mixture was vacuum-filtrated to remove all particles in the solution. The latter was analyzed by ICP-OES (IRIS Intrepid II XSP, Thermo Fisher, Waltham, MA, USA). The pH and conductivity were measured using a Mettler Toledo pH meter (SevenGo Duo Pro, Waltham, MA, USA).

### 2.4. Characterization of Geopolymer Samples

Compressive strength was performed after 28 days using a compression testing machine (Toni Technik, Berlin, Germany) according to EN 196-1:2016 described in [26]. The water absorption, porosity, and density were conducted according to the ASTM C–642 method and the Archimedes principle, respectively. Crystalline phases of all samples were determined by X-ray diffraction on an Empyrean PANalytical diffractometer (Malvern Panalytical Ltd., Malvern, UK) with a Ni filter transmitting the CuKα radiation (λkα1Cu = 1.540598 Å) produced at 40 mA and 40 kV. The infrared spectra curves were recorded in the range of 400–4000 cm^−1^ using an ATR method on Spectrum Two from (Buckinghamshire, UK), operating with a resolution of 4 cm^−1^. The microstructure was observed on a backscattered electron-scanning electron microscope (SEM) coupled with energy-dispersive X-ray spectroscopy (EDX) (Zeiss Gemini SEM 500 NanoVP microscope, Oberkochen, Germany), operating in low-vacuum mode with 15 kV acceleration voltage.

## 3. Results and Discussion

### 3.1. Stabilization Efficiency and Mechanism on Iron-Rich Laterite-Based Geopolymer

- Leaching of Cd and Cu from iron-rich lateritic soil-based geopolymer.

The potential for the leaching of Cd and Cu from the contaminated geopolymer matrix in different aqueous mediums, including water and hydrochloric acid and their effect on the binder chemistry, was carried out, as reported in Table 2. The results show that the type of aqueous medium in which the geopolymer is exposed affects the leachability of the metals. Indeed, the leached concentration of Cd and Cu in an acidic medium is higher than in water. The leached concentration of Cd is higher (>1 mg/L) in an acidic medium, which is beyond the threshold maximum recommended by the standard, while the leached concentration of Cu measured complies with regulation (100 mg/L for Cu and 1 mg/L for Cd) [6]. This can be related to the type of cadmium salt used since highly soluble salts, such as CdCl_2_, are easily dispersed throughout the geopolymer matrix, while sparingly soluble salt, such as CuSO_4_, remains segregated from the bulk of the binder [27]. Further, these observations also indicate that the mechanism of Cd solidification is through its precipitation as Cd(OH)_2_, knowing that its solubility at low pH is high [27]. Elsewhere, it is observed that the leached concentration of Al and Fe decreases from the contaminated geopolymers as compared to Gref. While for The presence of heavy metals, such as Na and Si, increases their concentration in both media. This observation is an indication of the stabilization mechanism occurring in iron-rich laterite-based geopolymer. The higher leached concentration of Na+ in contaminated geopolymers indicates that there is at least an ion exchange between heavy metals and Na+ in the geopolymer network to balance the negative charge due to the substitution of silica by alumina during the reaction [17]. Furthermore, the decrease of the leached concentration of Al and Fe from contaminated geopolymers, regarding the reference geopolymer, is proof of an efficient solidification of the heavy metals in this matrix. This means that the heavy metals formed chemical bonds with Al-O- and Fe-O- during geopolymerization and increased the strength of these bonds, reducing their depolymerization potential in leaching media [28].

- pH and conductivity of solutions after leaching tests.

**Figure 1:**
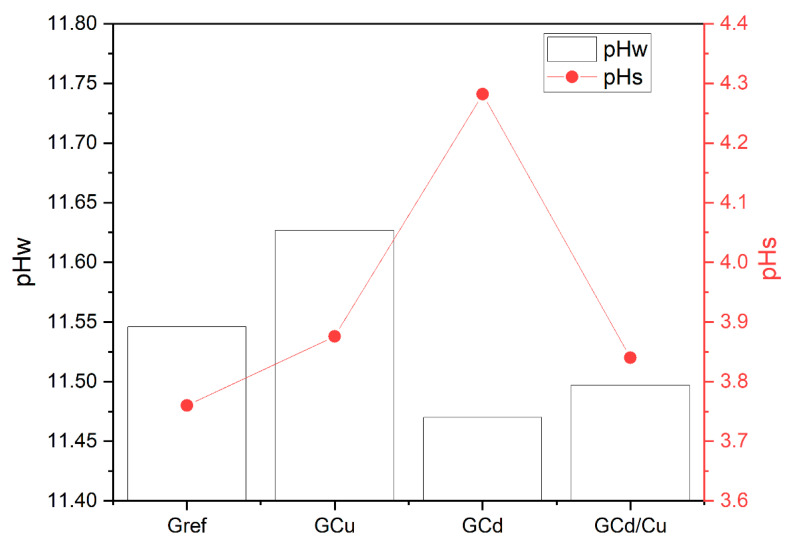
pH of the leachate from water (pHw) and hydrochloric acid (pHs).

Figure 1 presents the pH of the leachate obtained in distilled water (pHw) and hydrochloric acid (pHs). The pHw ranges from 11.47 to 11.63, and the pHs from 3.76 to 4.30. pHw and pHs are higher than the pH of the initial water (6.96) and hydrochloric acid (1.08) used. This is likely due to the leaching out of the alkali from the geopolymer in water, while in the acidic medium, it is linked to the acid–base reaction between the alkali from the geopolymer and protons (H+) from the acid solution. The pH of the leachate from the contaminated geopolymer GCu is higher, and the GCd is lower than the reference geopolymer in water. This can be explained by the sparse solubility of the copper salt (CuSO_4_·5H_2_O) in water as compared to CdCl_2_·H_2_O, which has contributed to keeping the pH high. However, in an acidic medium, the effect of the common ion effect in the case of GCd (since Cl^−^ is also issued by the HCl) would have reduced the solubility of the salt and fostered the acid–base reaction to increase the pH significantly. Thus, in the leachate containing Cu and Cd, one can assume that the pH change is mostly affected by the solubility of copper salt. This explains its high pH compared to GCd and lower pH than the GCu leachate obtained in water, while in the acid leachate, the pH from GCd is higher than GCd/Cu and GCu.

**Figure 2:**
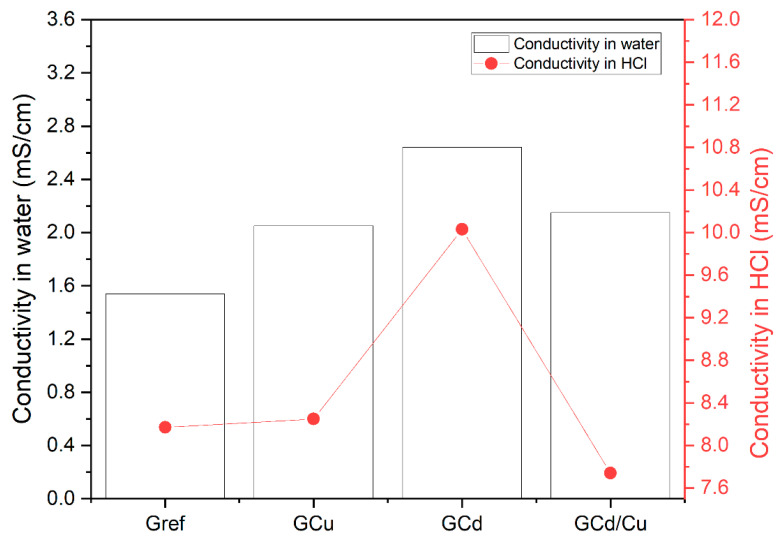
The conductivity of the leachate from water and hydrochloric acid.

It can be observed in Figure 2 that, independently of the leaching media, the conductivity of the leachate from GCd is highest, followed by GCd/Cu > GCu > Gref. The conductivity indicates the mobility of different ions contained in the solution and can be correlated with the total dissolved ions available in the leachate [29]. So, the higher the conductivity, the higher the content of ions in the solution. This confirms the statement on the high solubility of the cadmium salt as discussed earlier, which increased the conductivity of the resulting leachate. Moreover, the conductivity of the leachate obtained in an acidic medium is higher than the one in water. This result agrees with the data reported in Table 2, which shows a higher concentration of ions in acid solution than in water.

### 3.2. The Effect of Heavy Metals on the Mineralogy of Geopolymer

**Figure 3:**
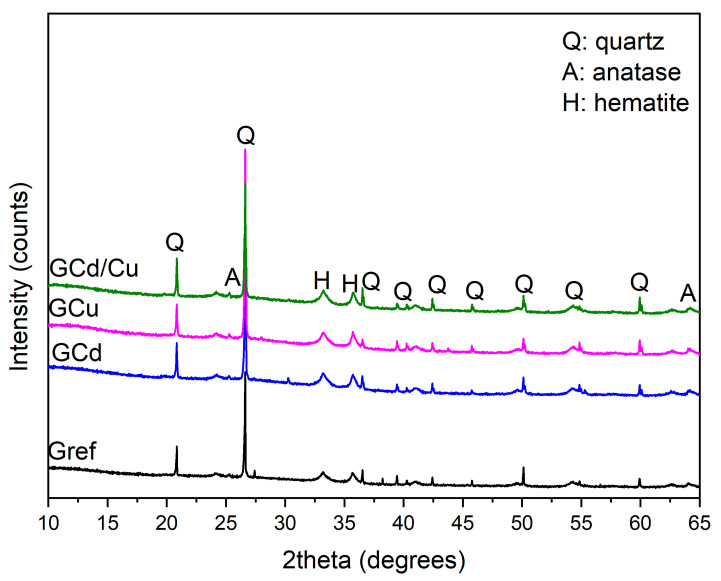
XRD patterns of reference and contaminated geopolymers.

**Figure 4:**
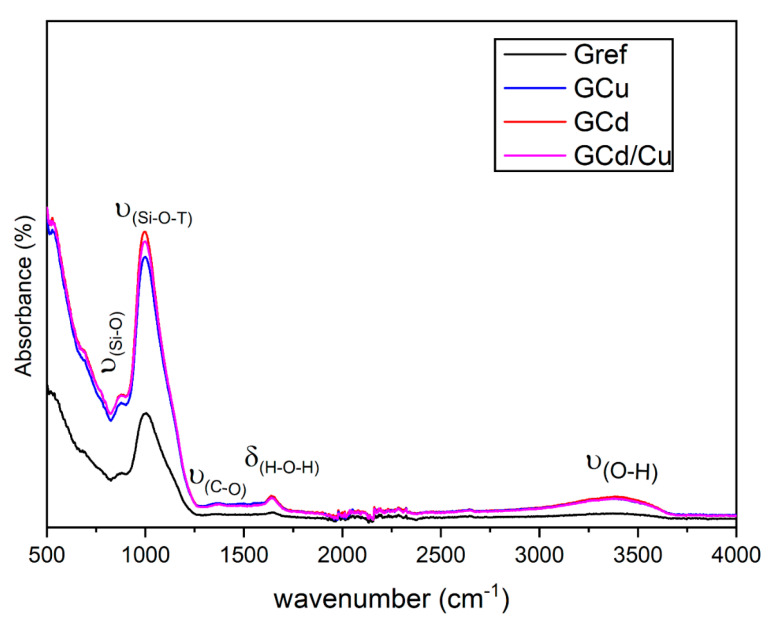
FTIR spectra of reference and contaminated geopolymers.

The XRD pattern of the reference geopolymer and the contaminated ones are presented in Figure 3. There are three major phases in all geopolymers, including hematite, quartz, and anatase. All the minerals present in Gref are still observed in the contaminated geopolymers. This infers that the heavy metals do not affect the mineralogy of the geopolymer and indicates that heavy metals are likely inserted in the amorphous geopolymer [28]. This also holds for the mineralogy after leaching in HCl and water (not shown here), where no considerable changes have been identified. The FT-IR spectra of the samples are depicted in Figure 4. The main change is observed in the band at around 1007 cm^−1^, corresponding to the stretching vibration of Si-O-T bonds (T = Si, Al, Fe), characteristic of the geopolymer binder. As observed, the intensity of that band is high in the contaminated geopolymers as compared to the reference geopolymer. This may be taken as being indicative of the effect of the heavy metal ions on the structure of the geopolymer network [27]. This change is more prominent in the spectrum of GCd than in GCu and GCd/GCu. This implies that the incorporation of cadmium affects the geopolymer structure significantly more than copper [30]. The absorption bands appearing at 3519 cm^−1^ and 1660 cm^−1^ are related to the stretching vibration of the O-H bond and the bending vibration of the H-O-H bond from water, respectively. The high intensity of the band at 3519 cm^−1^ in the contaminated geopolymers can also be an indication of the presence of the precipitated cadmium hydroxide and copper hydroxide since they are likely to be formed in the present system, as reported in previous work [31]. The band at 1371 cm^−1^ corresponds to the asymmetric stretching vibration of the C-O bonds of CO_3_^2−^ due to atmospheric carbonation on the geopolymer sample.

### 3.3. Hardened Characteristics of Contaminated Geopolymers

- Compressive strength and physical properties.

**Figure 5:**
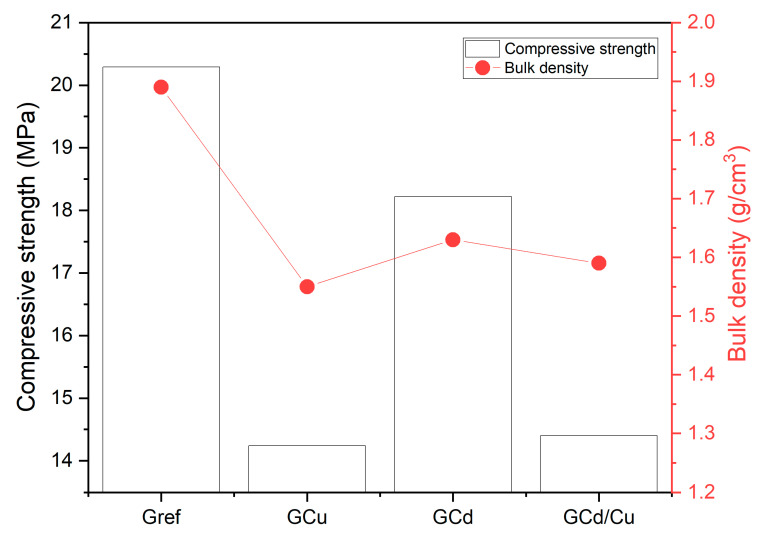
Compressive strength and bulk density of geopolymer samples.

**Figure 6:**
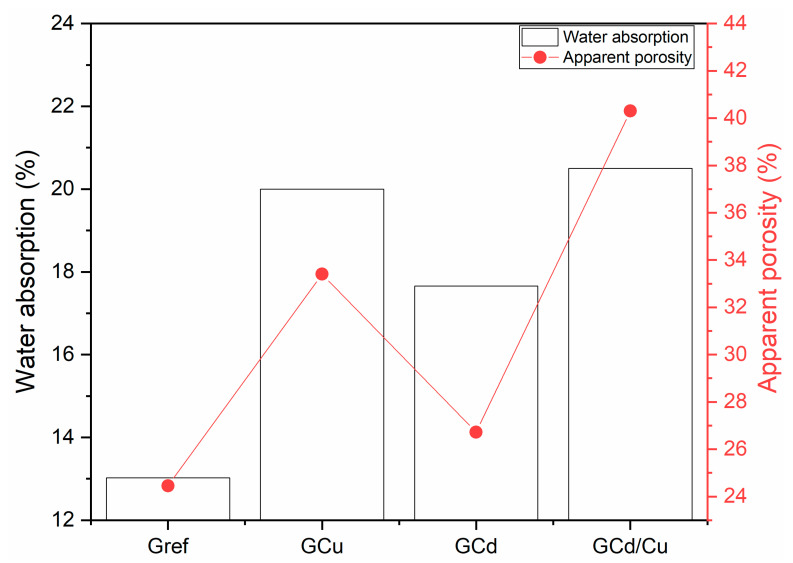
Apparent porosity and water absorption of geopolymer samples.

The compressive strength and bulk density of geopolymer samples are presented in Figure 5. The values obtained for compressive strength are 20.3, 14.3, 18.5, and 14.5 Mpa for Gref, GCu, GCd, and GCd/C, respectively. It is observed that the addition of Cd and Cu decreased the 28-day compressive strength. The same observation is performed for the bulk densities, where the values are: 1.9, 1.55, 1.63, and 1.6 g/cm^3^. This indicates that the heavy metals were integrated with the geopolymer network and created structural defects that led to a strength decrease. However, for Cd-contaminated geopolymer, the decrease observed is most likely related to the type of salt added. Since a report demonstrated that the addition of other salts, such as Cd(NO_3_)_2_, did not change the compressive strength [27]. Further, it was demonstrated that the strength decrease in CdCl_2_ might be attributed to the effects of the chloride contamination of the geopolymerization [32]. The highest strength decreases observed with Cu-contaminated geopolymer can be due to the way copper salt interacts during geopolymerization. Early in this work, it was reported that the heavy metal stabilization in this matrix also involves their precipitation as metal–hydroxide. The decrease of alkalinity due to the precipitation reaction likely hinders the dissolution rate of Si, Fe, and Al. Consequently, this slows down the growth of the geopolymer network skeleton, which reduces the compressive strength of the geopolymer [33]. This has also been emphasized in recent work, where it was demonstrated that the addition of ZnO during the synthesis of metakaolin-based geopolymer hinders the reaction and limits strength development [34]. The effect of these heavy metals on compressive strength can also be correlated with the trend of bulk density, which evolves Gref > GCd > GCu/Cu. The apparent porosity and water absorption presented in Figure 6 also agree with the evolution of compressive strength. In fact, the addition of heavy metals increased both the apparent porosity and the water absorption. The results yield 24, 34, 17, and 41% of porosity for Gref, GCu, GCd, and GCd/C, respectively, with water absorption of 12.5, 19.5, 17.5, and 20%.

- Capillarity water absorption.

**Figure 7:**
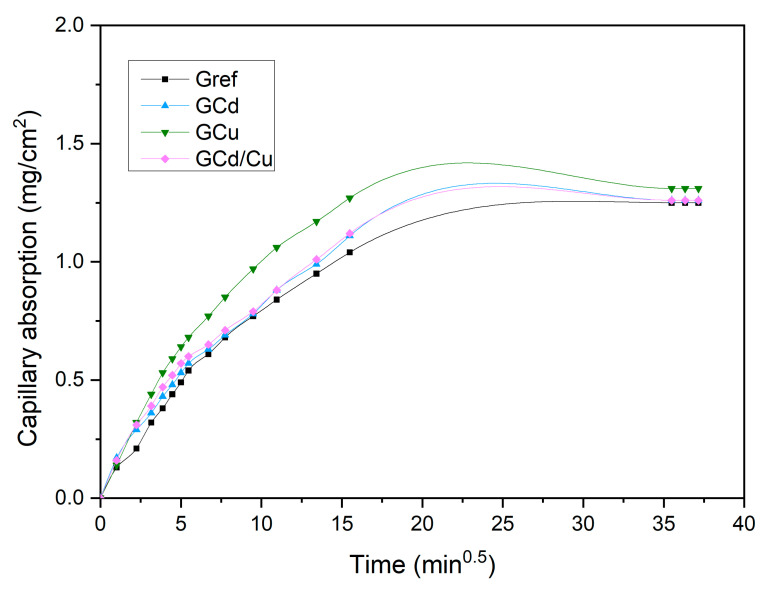
Capillary water absorption of geopolymer samples.

To explain the potential of a monolith geopolymer to resist leaching out of the stabilized heavy metals, the water penetration through a capillary pore has been carried out and reported in Figure 7. It can be observed that the curve is characterized by two trends, with the first one consisting of fast water penetration, while the second phase is steadily and mainly governed by the entrapped air diffusing out after the water saturation is reached. However, for all the samples, the water penetration rate is lower (0.1356, 0.1683, 0.1923, and 0.1872 mg/cm^2^·min^0.5^ for Gref, GCd, GCu, and GCd/Cu, respectively). This means that when exposed to an aggressive environment liable to leach out the stabilized contaminant, this process will happen very slowly over time. The tendency of the rate of water penetration observed among the reference and contaminated geopolymers agrees with the evolution of the porosity.

### 3.4. Microstructure

**Figure 8:**
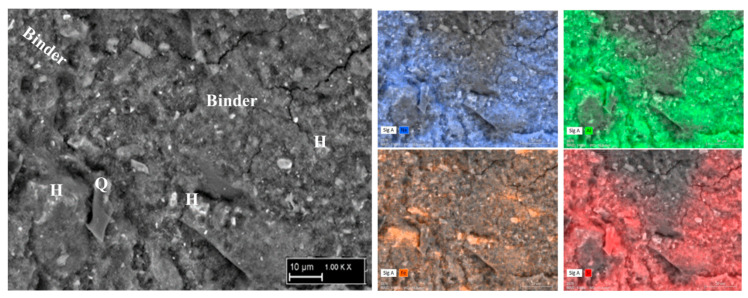
Microstructure and elemental map of reference geopolymer (H: hematite, Q: quartz).

**Figure 9:**
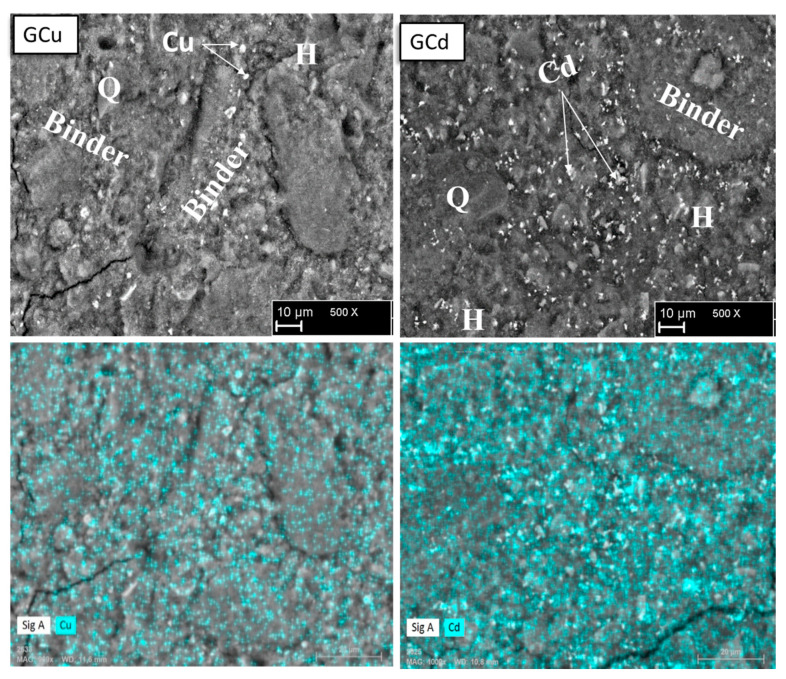
Microstructure of contaminated geopolymers with mapping showing the distribution of the corresponding heavy metals (H: hematite, Q: quartz).

**Figure 10:**
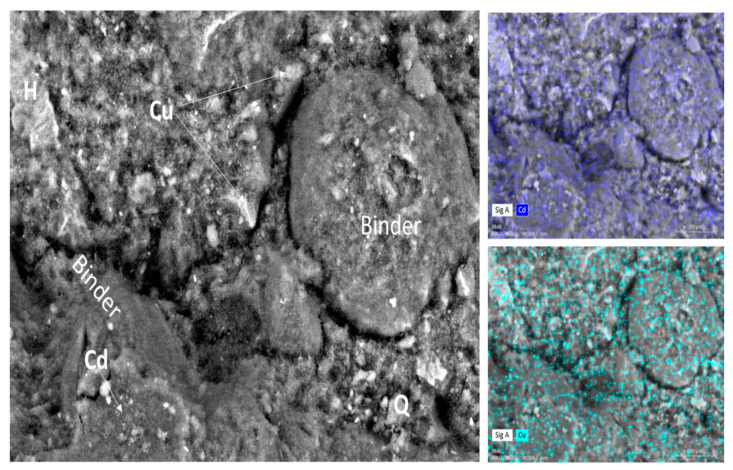
Microstructure of GCd/Cu with mapping showing the distribution of the corresponding heavy metals (H: hematite, Q: quartz).

**Figure 11:**
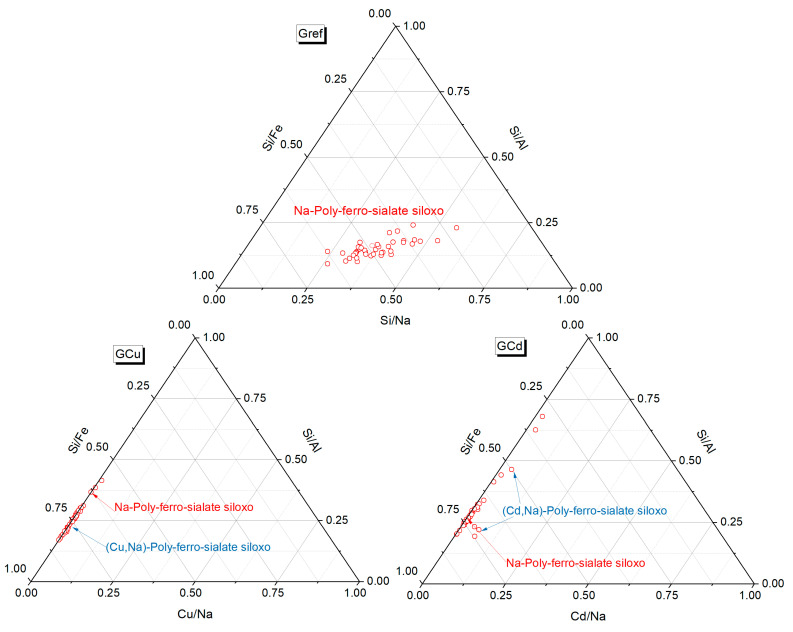
Ternary diagram showing the binder chemistry.

**Figure 12:**
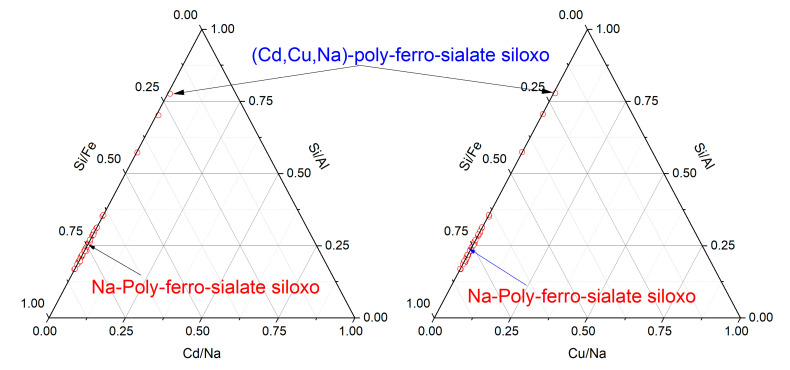
Ternary diagram showing the binder chemistry GCd/Cu.

The microstructure of samples Gref, GCd, and GCu and the representation of the different elements in the samples are highlighted in Figure 8 and Figure 9. The reference geopolymer shows a dense microstructure with shapeless phases. When heavy metals are added, the microcracks are more represented, which somehow explains the compressive strength decrease and porosity increase, as discussed earlier. The literature stated that the presence of more cracks with the addition of heavy metals in the structure of geopolymer can explain the effective solidification/stabilization of those heavy metals in the matrix [35]. The mapping of the distribution of all the elements present has been used to identify the behavior of heavy metals and to distinguish different features in the microstructure. In Figure 8, the brightest features of silicon and iron maps are identified as the non-reacted particles of quartz (Q) and hematite (H), respectively, whereas the non-bright area contains a homogeneous distribution of all the elements and corresponds to the binder. They have an average Si/Al atomic ratio < 2 and Si/Fe ranging from 2 to 7, which corresponds with Na-poly-ferro-sialate siloxo binder [25]. The microstructure and elemental maps of the contaminated geopolymers are shown in Figure 9. It is observed that Cu is uniformly distributed in all the areas of different samples constituting the binder. The microstructure also shows unreacted phases (hematite and quartz) along with cadmium, which appears as a bright part of the mapping. The presence of both metals simultaneously does not show any specific changes in their distribution within the binder, as observed in Figure 10. The presence of heavy metals in the binder shows that they have chemically reacted during geopolymerization. The analysis of the EDX composition of more than 40 spots through each contaminated geopolymer helped to identify the chemical composition of the binder and the changes brought by the addition of the heavy metals. Figure 11 and Figure 12 show that the composition of the reacted phases (binders) is modified by the presence of heavy metals. Initially, the main composition identified is Na-poly-ferro-sialate siloxo, which is present in all mixes and consists of the reaction of the iron-rich laterite with sodium silicate. This nomenclature means that the basic unit of the polymer is composed of iron, alumina, silica, and sodium. When Cu and Cd are added, either separately or simultaneously, the new binder chemistry is composed of (Cu,Na)-poly-ferro-sialate siloxo, (Cd,Na)-poly-ferro-sialate siloxo, and (Cu,Cd,Na)-poly-ferro-sialate siloxo, respectively. This indicates that Cu and Cd have chemically reacted during geopolymerization through the ion exchange process with sodium, which ascertains the stabilization mechanism stated earlier in this section.

## 4. Conclusions

In this work, iron-rich laterite-based geopolymers were prepared successfully from the alkaline activation process for the stabilization of heavy metals (Cd(II) and Cu(II)). It was found that the compressive strength and bulk density decrease with the addition of heavy metals when the apparent porosity and water absorption increase. Through the study of the leaching test, it was concluded that the stabilization efficiency of heavy metals is higher, and some factors, such as pH and conductivity, have a great influence. The leaching increases in an acidic medium and decreases in water. The FTIR and XRD realized on geopolymer indicated that the addition of heavy metals does not affect the crystal phases. The SEM observation shows a good distribution of heavy metals within the geopolymer matrix, suggesting a chemical and physical stabilization mechanism.

## Figures and Tables

**Table 1:** Mix compositions of reference and contaminated geopolymer matrix.

Sample Code	Calcined Laterite (g)	Ratio Liquid/Solid	Metal Salt Content (g)
Gref	300	0.6	0
GCd	300	0.6	3
GCu	300	0.6	3
GCd/Cu	300	0.6	1.5/1.5

Gref is the reference geopolymer without metals; GCd, GCu, and GCd/Cu are the contaminated geopolymers with the respective metals.

**Table 2:** Leaching test results in water and hydrochloric acid solution.

Heavy Metals	Leaching in Water				Leaching in HCl			
	Concentration (mg/L)							
	Gref	GCd	GCu	GCd/Cu	Gref	GCd	GCu	GCd/Cu
Al	2.95	1.63	2.16	2.95	491.70	28.63	361.30	382
Fe	0.36	0.17	0.17	0.16	0.78	0.55	0.48	0.50
Na	222.2	367.70	247	220.90	786.30	2377	1014	865.50
Si	20.44	23.10	31.09	21.60	7.12	37.66	56.18	58.85
Cd	0	0.017	0	0.024	0	51.68	0	17.31
Cu	0	0	0.03	0.03	0	0	19.89	7.49

## Data Availability

Data are contained within the article.
